# A Liquid-Metal Based Spiral Magnetohydrodynamic Micropump

**DOI:** 10.3390/mi8120365

**Published:** 2017-12-18

**Authors:** Xuyan Zhou, Meng Gao, Lin Gui

**Affiliations:** 1 Beijing Key Lab of Cryo-Biomedical Engineering and Key Lab of Cryogenics, Technical Institute of Physics and Chemistry, Chinese Academy of Sciences, 29 Zhongguancun East Road, Haidian District, Beijing 100190, China; xuyanzhou2016@gmail.com (X.Z.); mgao@mail.ipc.ac.cn (M.G.); 2 University of Chinese Academy of Sciences, 19 Yuquan Road, Shijingshan District, Beijing 100039, China

**Keywords:** microfluidics, magnetohydrodynamic, micropump, liquid metal

## Abstract

A liquid-metal based spiral magnetohydrodynamic (MHD) micropump is proposed in this work. The micropump was fabricated in a polydimethylsiloxane (PDMS)-glass hybrid microfluidic chip. This pump utilized two parallel liquid-metal-filled channels as electrodes to generate a parallel electrical field across the pumping channel between the two electrodes. To prevent contact and cross contamination between the liquid metal in the electrode channel and the sample fluid in the pumping channel, a PDMS gap was designed between the liquid metal and the sample fluid. To minimize the chip size, the parallel electrode and pumping channels were designed in a spiral shape. To test pumping performance, NaCl aqueous solution containing fluorescent particles (0.5 μm in diameter) was filled into the pumping channel as the working sample fluid. When a pair of identical magnets (0.4 T) was placed onto both top and bottom surfaces of the chip, the pump was able to drive the sample fluid at a flow velocity of 233.26 μm/s at 3000 V. The pump has no moving parts, and the electrodes are easily fabricated, making the pump suitable for miniaturization and integration into microfluidic systems.

## 1. Introduction

Lab-on-a-chip (LOC) is a technology that integrates biological and chemical applications, such as sample preparation, collection, reaction, separation, detection, and so on, into just one small chip [1]. Usually, LOC systems consist of various microfluidic channels. One of the key techniques of LOC is to manipulate the fluid in these microfluidic channels. As the “heart” of the whole microfluidic system, a micropump is widely used to drive the fluids in these microfluidic channels. Researchers have done lots of work on how to make micropumps more efficient and handy for use [2,3,4].

Micropumps are usually classified into two standard categories: mechanical micropumps and non-mechanical micropumps. Mechanical micropumps have moving parts inside, such as pneumatic membranes [5,6,7], piezoelectric elements, acoustic components [8], electrostatic coils [9], electrochemical bubbles [10], shape memory alloy, and ultrasonic excitation [11]. These moving parts decrease the life span and operational stability of the pump. Furthermore, they also require a complicated fabrication process that is of high cost. In contrast, non-mechanical micropumps, which have no moving parts, are more stable and reliable in terms of performance. In recent decades, many non-mechanical micropumps have been proposed and demonstrated, such as electroosmotic micropumps [12], surface tension micropumps [13], electro hydrodynamic micropumps [14], electromagnetic hydrodynamic micropumps [15], laser-driven micropumps [16], and so on. Among them, electric control is an efficient and convenient method to drive the microfluid. Electroosmotic micropumps and electromagnetic hydrodynamic micropumps possess the merits of both non-mechanical micropumps and electric control methods. Although electroosmotic micropumps are very convenient for microfluidic use, they are not suitable for driving conductive sample fluids such as electrolytic aqueous solutions, which are widely used in microfluidics. The magnetohydrodynamic (MHD) micropump is a good choice for these electrolytic aqueous solutions. It has been widely used to drive conductive sample fluids in many microfluidic applications, including mixing [17], liquid chromatographs [18], and integrated fluidic networks [19].

In 2000, Jang et al. fabricated a MHD micropump on a bulk-etched silicon wafer to drive seawater [20]. This pump was able to drive at a flow rate of 63 μL/min, with a maximum pressure head of 18 mm when 1.8 mA electrical current and 0.44 T magnetic field were applied. However, due to the contact of the seawater with the electrode, the electrolysis was generated near the solid metal electrodes, leading to bubble formation, Joule heating, and electrode corrosion. Many attempts have been made to solve these problems in recent years. AC current at high frequency is a good choice for electrolysis-free pumping, but it will drive fluid with pulse flows [18,21]. DC magnetohydrodynamic micropumps with relatively low voltage are another efficient choice for avoiding electrolysis, but offer continuous flows.

With the development of microfluidic technologies, more and more microfluidic systems need flexible pumping components. However, most of the MHD micro-pumps reported are fabricated with silicon [20,21], ceramic substrates [22], low-temperature co-fired ceramic tapes (LTCC) [17,19], glass [15], photosensitive glass [23], and acrylic plastic [24]. Solid metal (Au, platinum, copper)-based wires and membranes are usually used as electrodes for MHD micropumps. The pumping channels and electrodes for these pumps are fabricated with hard materials, and are not suitable for flexible applications. In 2011, a MHD micropump fabricated with flexible polydimethylsiloxane (PDMS) material for a mixer was first proposed and demonstrated by Ho-Jin Kang and Bumkyoo Choi [25]. Unfortunately, the electrodes for this pump were still fabricated with a solid metal (Au membrane patterned on the chip substrate).

Electrode material is becoming a key factor that constrains the applications of MHD micropumps in the fields of stretchable electronics or implantable micro devices. Since the favorable properties of liquid metal in micro channels have been reported in recent decades, liquid metal has gained significant popularity among microfluidic researchers. The liquid metal alloys (GaInSn or gallium-base alloy) can be injected into micro channels rapidly when enough pressure is applied to the inlet of the channels, and can then maintain structural stability in the channels once the extra pressure is relieved [26,27]. Due to its high electrical conductivity, high surface tension, low toxicity, and low viscosity, liquid metal has been widely used for electrodes and antennas [28,29,30] in micro devices including pressure sensors [31,32,33], electroosmotic pumps [2], microwave circuits [30], and liquid and gas-phase VOC detectors [34].

In addition, a large number of numerical simulations have been carried out to study the performance of MHD pumps [35,36]. Recently, the in-depth improvement of numerical simulations for electrochemical MHD studies has provided new insight into the development of microfluidic devices [37,38].

**Figure 1:**
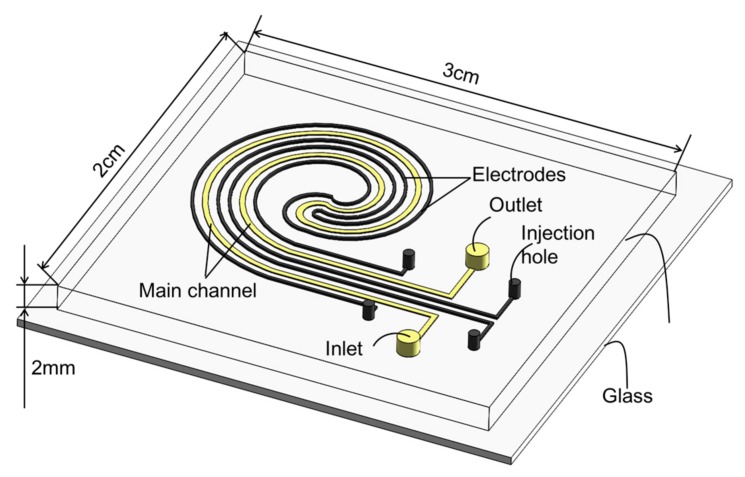
Schematic of the polydimethylsiloxane (PDMS)-glass microfluidic chip for the liquid-metal-based magnetohydrodynamic (MHD) micropump.

**Figure 2:**
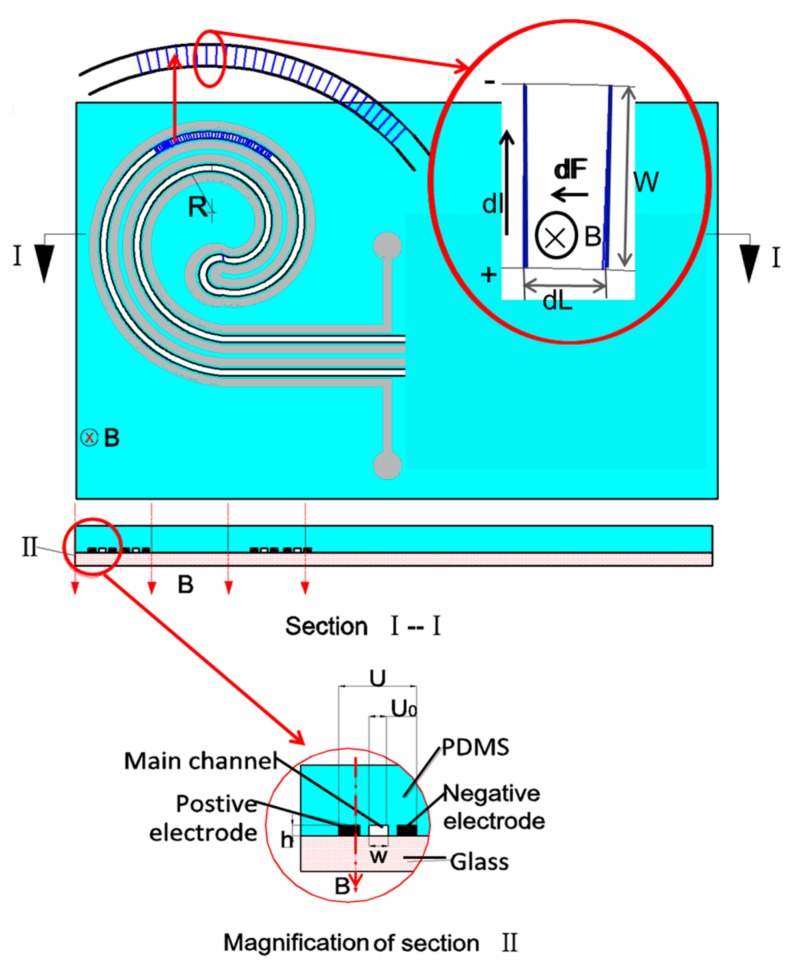
Working principles of the liquid-metal-based MHD pump.

In this paper, a liquid-metal-based spiral MHD micropump using PDMS material is proposed and demonstrated. It consists of a spiral pumping channel with two parallel liquid-metal-filled electrodes located on both sides of the pumping channel at the same horizontal level (as shown in Figure 1 and Figure 2). Injection of liquid metal into the PDMS microchannels to form flexible electrodes makes the fabrication process of the pump relatively simple and cost effective. Additionally, the liquid metal electrodes encapsulated with PDMS are able to prevent cross-contamination between the liquid metal and the sample fluid, thus eliminating electrolysis. The PDMS gap between the liquid metal electrode and the pumping channel in this pump is weakly conductive [2]. Thus, when a certain voltage is applied to both liquid metal electrodes, in addition to the vertical magnetic field through the pumping channel, an electrolyte solution will be driven to flow along the pumping channel under the Ampère’s force. In this work, we will fabricate this pump and test the pumping performance of the pump in detail.

## 2. Theory

When a charged conductor is placed in an external magnetic field (B), the interaction between the magnetic field and the electric current results in Ampère’s force. In the case of magnetohydrodynamics, or MHD, the conductor is a fluid. Ampère’s force, in turn, can be used to pump and manipulate the fluid [39]. That is the working principle of the MHD micropump. An electrically conductive or weakly electrically conductive solution that fills the pumping microchannel will be driven by Ampère’s force. This is suitable for many of the working fluids in biomedical applications, such as buffer solutions.

Figure 1 shows the microfluidic chip for the liquid-metal-based MHD micropump. The chip is 3 cm long, 2 cm wide, and 3 mm thick (1 mm thick glass slide, 2 mm thick PDMS slab). The two parallel spiral micro electrode channels are filled with liquid metal at both sides of the main pumping channel at the same horizontal level. Because the electrode channels and the pumping channel are separated by a PDMS gap (50 μm), cross-contamination between the liquid metal and the working fluid can be prevented. For the convenience of the liquid-metal injection, both injection holes of the electrode channel are placed 3 mm away from the main pumping channel.

According to the law of Ampère’s force, the Ampère’s force is perpendicular both to the magnetic field and the electric current in the channel. Electric current *I* is the current across the channel, and is supplied by the voltage source. In our work, the total potential *U* is applied to the liquid metal electrode. As shown in Figure 2, because of the existence of PDMS gap between the electrode and the pumping channel (Main channel), the voltage *U*_0_ applied to the working fluid is less than the total potential *U*. The magnetic field strength B is supplied by a pair of magnets.

In our paper, the pumping channel is designed in a spiral shape. We assume the whole pumping channel to be equally divided into infinite small sections to analyze the force as shown in Figure 2. For each section, the channel length is *dL*. If channel width *W* is far less than the spiral channel radius *R*, all the small sections can be treated as rectangles. Therefore, the Ampère’s force *dF* induced in the small section can be written as:

(1) dF=BdIW

(2) $$dI=\frac{U_{0}}{\sigma \frac{W}{dL\cdot h}}=\frac{U_{0}h}{\sigma W}dL$$

where, σ is the resistivity of the electrolyte aqueous solution and *h* is the microchannel height. Then:

(3) $$dF=B\frac{U_{0}}{\sigma }hdL$$

Then, the Ampère’s force *F* in the whole channel can be expressed as:

(4) $$F=\int dF=\int B\frac{U_{0}}{\sigma }hdL=B\frac{U_{0}}{\sigma }hL$$

As shown in Equation (4), the driving force is proportional to the magnetic flux density *B*, the voltage *U*_0_, the channel height *h* and the pumping length *L*. In this work, we will demonstrate the impact of these parameters on the pumping performance of the micropump. Obviously, the working fluid must be electrically conductive, or at least weakly electrically conductive, to allow the electric current to be generated. Fortunately, most of the working fluids in biomedical applications, such as the buffer solutions, are electrically conductive.

## 3. Experimental Details

### 3.1. Chip Fabrication

The microfluidic chips for MHD micropumps were fabricated by using soft-lithography. SU-8 2050 and SU-8 2075 (MicroChem Corp., Westborough, MA, USA) were used to fabricate 50 μm- and 150 μm-high micro channel patterns on silicon wafers, respectively. PDMS (mixture of base and curing agent at 10:1 per weight, Dow Corning Corp. Midland, MI, USA) was poured onto the wafers to transfer the channel patterns. Then, the patterned PDMS slab was bonded irreversibly with glass slide (75 mm × 50 mm × 1 mm) using air plasma (plasma cleaner, YZD08-2C, Tangshan Yanzhao Technology, Tangshan, China). Although we used a glass slide in the pump for the convenience of the observation in microscope, the glass is not a prerequisite, and we could fabricate this pump using pure PDMS in the future.

GaIn_20.5_Sn_13.5_(66 wt % Ga, 20.5 wt % In, 13.5 wt %; Shanxi Zhaofeng Gallium Co., Ltd., Shanxi, China) was injected into the electrode channels to fabricate electrodes using a syringe pump. If enough pressure is applied to the inlet of the channels, the liquid metal can be injected into the microchannels rapidly, and will remain in the channels even if the extra pressure is relieved because of its surface oxidation [26]. Then, copper wires (200 μm in diameter) were inserted into the injection holes as external wires to connect the high-voltage power supply. To fasten the copper wire, the joint between the copper wire and the liquid metal injection hole was sealed using a package adhesive sealant (705 RTV Transparent Silicone Rubber, Kangda Chemical Co., Ltd., Liyang, China).

### 3.2. Velocity Measurement

**Figure 3:**
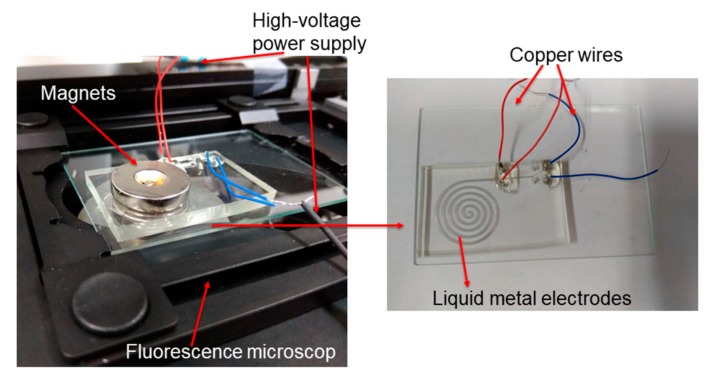
Experimental set-up for measurement of the pumping velocity of the liquid-metal-based MHD pump.

Figure 3 shows the experimental setup for the pumping velocity measurement of the pump. A pair of ring-shaped Rubidium Iron Boron (RbFeB) magnets was placed onto the top and bottom surfaces of the chip respectively to offer a uniform magnetic field inside the pumping channels. The magnetic field strength was 0.4 T (measured by Gaussmeter Model GM2, AlphaLab, Inc., Salt Lake City, UT, USA).

A fluorescence microscope (Axio Observer Z1, Carl Zeiss, Jena, Germany) was used to monitor the tracing particles in the sample fluid flow. A high-voltage sequencer (HVS448 6000D, LabSmith, Inc., Livermore, CA, USA) was used to provide high voltages for the pump. Then, NaCl fluorescent solution (mixture of NaCl solution and fluorescent particles at a 1000:1 ratio per volume) (Ex 542 nm, Em 612 nm, 1% solids, Duke Scientific Corporation, Palo Alto, CA, USA) was injected into the pumping channel for testing.

## 4. Results and Discussion

**Figure 4:**
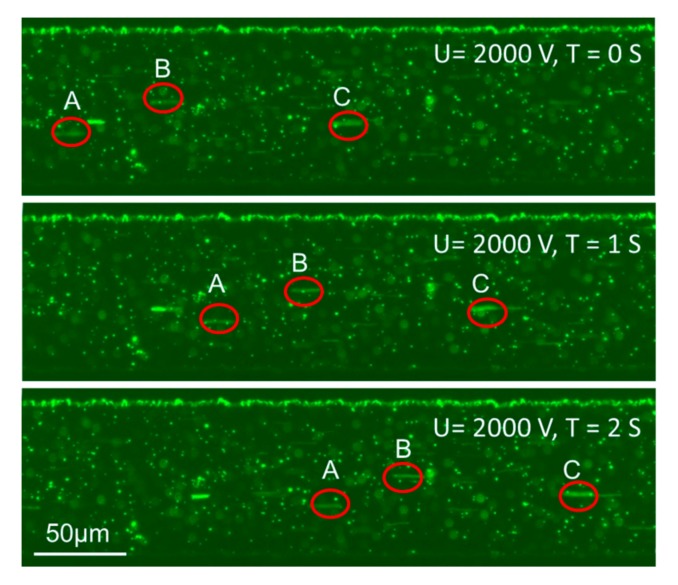
Sequential photographs of 0.5 μm polystyrene particles in 200 μm-wide microchannels. The average velocities of the three particles measured in μm/s were: A = 109, B = 105, C = 115.

Figure 4 shows three sequential photographs of 0.5 μm polystyrene particles in 200 μm-wide micro channels at 2000 V. In this pump, the spiral pumping channel was 200 μm wide and 12 cm long. The non-pumping channel was 5 mm long. All channels were 150 μm high. The PDMS gap between the electrode channel and the pumping channel was 50 μm. A 36-second movie describing the movement of particles in the pumping flows at 1800–2400 V is included in the Video S1 in Supplementary Materials. A, B, C, and the circle marks represent three random tracing fluorescent particles. The average velocities of the targeted particles were: 109 μm/s, 105 μm/s, and 115 μm/s, respectively. Then, we were able to obtain the pumping flow velocity of the pump (109.67 μm/s) by averaging the velocities of the targeted particles above.

To investigate the influence of chip size and fluid properties on the pumping performance, parametric studies were performed, including pumping channel length (6 cm, 9 cm, and 12 cm), pumping channel height (100 μm, 120 μm and 150 μm), and saline solution concentration ω (0.1% NaCl, 0.3% NaCl, 0.9% NaCl). The MHD pump ran stably and continuously for more than five hours. Throughout the whole experiment, the electric current varied from 30 μA to 110 μA within a voltage range from 1000 V to 3000 V.

**Figure 5:**
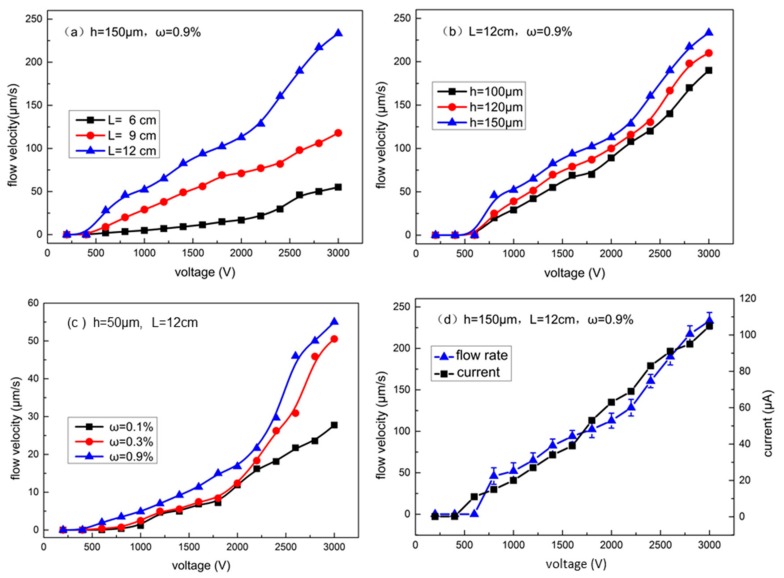
Experimental results of parametric studies. (**a**) Flow velocity as a function of voltage applied with different pumping zone channel lengths (the non-pumping channel was 5 mm long. All channels were 50 μm high); (**b**) Flow velocity as a function of voltage with different channel heights; (**c**) Flow velocity as a function of voltage with different saline solution concentration (ω); (**d**) Flow velocity and the average current as functions of different voltages applied to the same pump.

Figure 5a shows the fluid flow velocity as a function of applied voltage with different channel length in the pumping zone. Three pumping channel lengths were considered: 6 cm, 9 cm and 12 cm. The channel width was 200 μm and the PDMS gap between the electrode and pumping channels was 50 μm. The channel length in the non-pumping zone was 5 mm, and its height was 150 μm. 0.9% NaCl aqueous solution was used as the working fluid in this work. In order to make the footprint as small as possible, we designed the flow path in a spiral shape. To increase the length of the runner, the number of circles could be increased. As shown in Figure 5a, the micro pump with longer pumping channels was able to drive the fluid at higher velocities. The pumps with 12-cm and 9-cm pumping channels were able to drive the fluid flows with velocities of 28 μm/s and 9 μm/s at 600 V, respectively. However, the pump with the 6-cm pumping channel was not able to drive the flow when the applied voltage was 600 V. As shown in Equation (4), the driving force is proportional to the length of the pumping channel; the longer the channel is, the stronger the driving force. With larger driving force and the same flow resistance with respect to the non-pumping channel, the pumping rate will increase. Furthermore, in our work, the whole pumping channel, together with the PDMS gaps, works as a resistor. Increasing the length will decrease the equivalent resistance and increase the current. Therefore, increasing the length of the channel will increase the pumping rate. This is the reason why we designed the micro pumps with longer pumping channels in a spiral shape. However, a pumping channel that is too long will extend the pumping area. 12 cm is recommended as the longest pumping length for a 1 cm^2^ pumping area. The maximum possible pumping velocity can reach up to 233.26 μm/s, with a corresponding flow rate of 0.411 uL/min, before the PDMS gap is broken at 3000 V.

Figure 5b shows the flow velocity as a function of voltage with different channel heights. The pumping channel was 200 μm wide and 12 cm long. The non-pumping channel was 5 mm long. The PDMS gap was 50 μm. Three channel heights were considered: 100 μm, 120 μm and 150 μm. As shown in Figure 5b, the pump with a 150 μm-high channel was able to reach the highest flow velocity (the blue curve in Figure 5b). Higher pumping channels could decrease the equivalent electrical resistance, and greater Ampère’s force could be produced due to the greater current density with the application of a certain voltage.

Figure 5c shows the flow velocity as a function of voltage with different saline solution concentrations (mass ratio ω: 0.1%, 0.3% and 0.9%). Higher ion concentrations could raise current intensity with application of a certain voltage. The flow velocity will increase with the increment of the saline solution density.

Figure 5d shows the flow velocity and the average current as functions of different voltages applied to the same pump. Overall, the current density increases with the voltage. The increase of the flow velocity lags slightly behind that of the voltage due to the presence of the non-pumping zone and its flow resistance. The minimum driving voltage is about 600 V. As shown in Figure 5d, although the liquid metal does not contact with the working fluid, a small current is still observed in this pump. That is to say, although PDMS is insulated, a small current is still generated in the PDMS membrane between the liquid metal electrode and the working fluid, which was also observed in our former work [2]. The further reasons for this are still unclear, and are beyond the scope of this work.

## 5. Conclusions

The MHD micro pump described in this work showed strong driving performance. On the one hand, it was able to offer stable high-volume output and could be precisely controlled. On the other hand, non-contact liquid-metal electrodes are able to avoid the hydrolysis reaction and cross-contamination between the liquid metal and the sample fluid. Thus, this liquid-metal-based MHD pump was able to successfully eliminate bubble generation and electrode erosion. Furthermore, because the current generated in the pump was very low, the Joule heat in this micropump could be neglected. This MHD micro pump has the merits of simple structure, easy fabrication, low cost, high stability and precise control, with no moving parts. The utilization of liquid metal electrodes could help the miniaturization and integration of the pump into microfluidic systems. We just need to add the spiral pumping channel and electrode channel when we design the whole microfluidic chip; additionally, all the pumps may share the same magnet. In the future, the pump could have great potential in a wide variety of applications, such as microfluidic analysis and MEMS chip cooling.

There are still some factors that can be improved in the future for further applications, such as replacing the permanent magnets with smaller micro electromagnets, shrinking the chip size, etc. Meanwhile, the driving voltage could also be lowered by reducing the thickness of the PDMS layer between the electrodes and the pumping fluid. If the PDMS layer were thinner, the electric resistance of the PDMS layer would decrease sharply, and the voltage falling on this PDMS layer would also decrease with it. Therefore, for the same electric current (i.e., the same pumping capacity), the thinner the PDMS gap is, the smaller the driving voltage. The goal of this work was to propose a new pumping method by using liquid metal electrodes. Extensive work can now be undertaken to improve this pump in the future, according to the specific requirements of further applications.

## Supplementary Materials

The following are available online at www.mdpi.com/2072-666X/8/12/365/s1, Video S1: Flow in microchannel driven by a magnetohydrodynamic micropump.

## References

[1] Mark D., Haeberle S., Roth G., Von Stetten F., Zengerle R. (2010). Microfluidic Lab-on-a-Chip Platforms: Requirements, Characteristics and Applications. Microfluidics Based Microsystems.

[2] Gao M., Gui L. (2014). A handy liquid metal based electroosmotic flow pump. Lab Chip.

[3] Rupp J., Schmidt M., Munch S., Cavalar M., Steller U., Steigert J., Stumber M., Dorrer C., Rothacher P., Zengerle R. (2012). Rapid microarray processing using a disposable hybridization chamber with an integrated micropump. Lab Chip.

[4] Wang A.B., Hsieh M.C. (2012). Unveiling the missing transport mechanism inside the valveless micropump. Lab Chip.

[5] Tseng H.Y., Wang C.H., Lin W.Y., Lee G.B. (2007). Membrane-activated microfluidic rotary devices for pumping and mixing. Biomed. Microdevices.

[6] Yang Y.N., Hsiung S.K., Lee G.B. (2009). A pneumatic micropump incorporated with a normally closed valve capable of generating a high pumping rate and a high back pressure. Microfluid. Nanofluid..

[7] Chen H., Cornwell J., Zhang H., Lim T., Resurreccion R., Port T., Rosengarten G., Nordon R.E. (2013). Cardiac-like flow generator for long-term imaging of endothelial cell responses to circulatory pulsatile flow at microscale. Lab Chip.

[8] Wixforth A. (2006). Acoustically driven programmable microfluidics for biological and chemical applications. J. Assoc. Lab. Autom..

[9] Xie J., Shih J., Lin Q., Yang B., Tai Y.C. (2004). Surface micromachined electrostatically actuated micro peristaltic pump. Lab Chip.

[10] Böhm S., Timmer B., Olthuis W., Bergveld P. (2000). A closed-loop controlled electrochemically actuated micro-dosing system. J. Micromech. Microeng..

[11] Xu J., Attinger D. (2007). Control and ultrasonic actuation of a gas–liquid interface in a microfluidic chip. J. Micromech. Microeng..

[12] Wang X., Cheng C., Wang S., Liu S. (2009). Electroosmotic pumps and their applications in microfluidic systems. Microfluid. Nanofluid..

[13] Resto P.J., Berthier E., Beebe D.J., Williams J.C. (2012). An inertia enhanced passive pumping mechanism for fluid flow in microfluidic devices. Lab Chip.

[14] Sawane Y.B., Datar S., Ogale S.B., Banpurkar A.G. (2015). Hysteretic DC electrowetting by field-induced nano-structurations on polystyrene films. Soft Matter.

[15] Homsy A., Koster S., Eijkel J.C.T., Berg A., Lucklum F., Verpoorte E., Rooij N.F. (2005). A high current density DC magnetohydrodynamic (MHD) micropump. Lab Chip.

[16] Chen Y., Wu T.H., Chiou P.Y. (2012). Scanning laser pulses driven microfluidic peristaltic membrane pump. Lab Chip.

[17] Bau H.H., Zhong J., Yi M.Q. (2001). A minute magneto hydro dynamic (MHD) mixer. Sens. Actuators B Chem..

[18] Eijkela J.C.T., Daltonb C., Haydenb C.J., Burtb J.P.H., Manza A. (2003). A circular ac magnetohydrodynamic micropump for chromatographic applications. Sens. Actuators B Chem..

[19] Bau H.H., Zhu J.Z., Qian S.Z., Xiang Y. (2003). A magneto-hydrodynamically controlled fluidic network. Sens. Actuators B Chem..

[20] Jang J., Lee S.S. (2000). Theoretical and experimental study of MHD (magnetohydrodynamic) micropump. Sens. Actuators A Phys..

[21] Lemoff A.V., Lee A.P. (2000). An AC magnetohydrodynamic micropump. Sens. Actuators B.

[22] Zhong J., Yi M., Bau H.H. (2002). Magneto hydrodynamic (MHD) pump fabricated with ceramic tapes. Sens. Actuators A Phys..

[23] Kim C.T., Lee J., Kwon S. (2014). Design, fabrication, and testing of a DC MHD micropump fabricated on photosensitive glass. Chem. Eng. Sci..

[24] Ito K., Takahashi T., Fujino T., Ishikawa M. (2014). Influences of Channel Size and Operating Conditions on Fluid Behavior in a MHD Micro Pump for Micro Total Analysis System. J. Int. Counc. Electr. Eng..

[25] Kang H.J., Choi B. (2011). Development of the MHD micropump with mixing function. Sens. Actuators A Phys..

[26] Dickey M.D., Chiechi R.C., Larsen R.J., Weiss E.A., Weitz D.A., Whitesides G.M. (2008). Eutectic Gallium-Indium (EGaIn): A Liquid Metal Alloy for the Formation of Stable Structures in Microchannels at Room Temperature. Adv. Funct. Mater..

[27] So J.H., Dickey M.D. (2011). Inherently aligned microfluidic electrodes composed of liquid metal. Lab Chip.

[28] Cheng S., Boczkowska A. (2012). Advanced Elastomers: Technology, Properties, and Application.

[29] Li G., Wu X., Lee D.-W. (2015). Selectively plated stretchable liquid metal wires for transparent electronics. Sens. Actuators B Chem..

[30] Zandvakili M., Honari M.M., Mousavi P., Sameoto D. (2017). Gecko-Gaskets for Multilayer, Complex, and Stretchable Liquid Metal Microwave Circuits and Antennas. Adv. Mater. Technol..

[31] Shin H.-S., Ryu J., Majidi C., Park Y.-L. (2016). Enhanced performance of microfluidic soft pressure sensors with embedded solid microspheres. J. Micromech. Microeng..

[32] Ponce Wong R.D., Posner J.D., Santos V.J. (2012). Flexible microfluidic normal force sensor skin for tactile feedback. Sens. Actuators A Phys..

[33] Jung T., Yang S. (2015). Highly Stable Liquid Metal-Based Pressure Sensor Integrated with a Microfluidic Channel. Sensors.

[34] Kim M., Alrowais H., Kim C., Yeon P., Ghovanloo M., Brand O. (2017). All-soft, battery-free, and wireless chemical sensing platform based on liquid metal for liquid and gas-phase VOC detection. Lab Chip.

[35] Wang P.J., Chang C.Y., Chang M.L. (2004). Simulation of two-dimensional fully developed laminar flow for a magneto-hydrodynamic (MHD) pump. Biosens. Bioelectron..

[36] Sen D., Isaac K.M., Leventis N., Fritsch I. (2011). Investigation of transient redox electrochemical MHD using numerical simulations. Int. J. Heat Mass Transf..

[37] Isaac K.M., Gonzales C., Sen D. (2014). Modeling of redox electrochemical MHD and three-dimensional CFD simulations of transient phenomena in microfluidic cells. Microfluid. Nanofluid..

[38] Yuan F., Isaac K.M. (2017). A study of MHD-based chaotic advection to enhance mixing in microfluidics using transient three dimensional CFD simulations. Sens. Actuators B Chem..

[39] Hughes M., Pericleous K.A., Cross M. (1994). The CFD analysis of simple parabolic and elliptic MHD flows. Appl. Math. Model..

